# Sensitivity to cisplatin in primary cell lines derived from human glioma correlates with levels of EGR-1 expression

**DOI:** 10.1186/1475-2867-11-5

**Published:** 2011-03-02

**Authors:** Antonella Calogero, Antonio Porcellini, Vincenza Lombari, Cinzia Fabbiano, Antonietta Arcella, Massimo Miscusi, Donatella Ponti, Giuseppe Ragona

**Affiliations:** 1 Department of Medical-surgical Science and Biotechnologies, University of Rome "Sapienza", Corso della Repubblica 79, 04100 Latina, Italy; 2 University of Molise, 86100 Campobasso, Italy; 3 IRCCS Neuromed, 86077 Pozzilli, Italy

## Abstract

**Background:**

Less than 30% of malignant gliomas respond to adjuvant chemotherapy. Here, we have asked whether variations in the constitutive expression of early-growth response factor 1 (EGR-1) predicted acute cytotoxicity and clonogenic cell death *in vitro*, induced by six different chemotherapics.

**Materials and methods:**

Cytotoxicity assays were performed on cells derived from fresh tumor explants of 18 human cases of malignant glioma. In addition to EGR-1, tumor cultures were investigated for genetic alterations and the expression of cancer regulating factors, related to the p53 pathway.

**Results:**

We found that sensitivity to cisplatin correlates significantly with levels of EGR-1 expression in tumors with wild-type *p53/INK4a/p16 *status.

**Conclusion:**

Increased knowledge of the mechanisms regulating EGR-1 expression in wild-type *p53/INK4a/p16 *cases of glioma may help in the design of new chemotherapeutic strategies for these tumors.

## Introduction

Malignant brain tumors of glial origin are highly invasive and poorly sensitive to anti-proliferative drugs, with only 20-30% of patients responding to chemotherapy. The biological basis of drug resistance in these tumors is complex, being dependent to some extent on the genetic make-up of the tumor. The prognostic value of molecular markers has been investigated either retrospectively, in patients treated with standard therapy, or in tumor cells cultured *in vitro *and exposed to different chemotherapics, but no clear results have emerged [1]. The role of *p53 *gene status [2], the presence of deletions in the *INK4a/INK4b *locus coding for the tumor suppressors and cell cycle regulators p16, p15 and p14ARF [3], the MGMT (O^6^-methylguanine DNA methyltransferase) levels [4] and the levels of expression for several players and regulators of apoptosis [5] were all studied to predict the response of the tumor to specific drugs. The rationale of these studies was that tumor cells react to the genotoxic insult by p53-dependent cell cycle arrest, or by undergoing apoptosis [6]. However, from these studies none of these factors, except MGMT, emerged as a major determinant of chemoresistance [7].

Many genes are found to be defective and others are deregulated in gliomas [8]. We have recently found that EGR-1 expression is downregulated in malignant gliomas [9]. EGR-1 encodes a nuclear transcription factor responsible for the regulation of cell differentiation and proliferation of several cell lineages, in response to external stimuli. By regulating, either positively or negatively, target genes such as TGF-β, cyclin D1, c-jun, PTEN, p53 and p21, EGR-1 decreases cell proliferation, carrying out tumor suppressive functions in several tumor types including gliomas [10,11]. In addition, some authors have found that its expression is associated with enhanced patient survival [12,13]. We also observed that EGR-1 is less expressed in tumors and tumor-derived primary cell lines carrying wild type copies of p53 gene compared to those carrying p53 mutated copies [14]. It has been shown that EGR-1 is required for the function of p53, since it acts as an upstream regulator of the p53 tumor suppressor pathway [15]. In turn, overexpression of mutant p53 activates EGR-1 expression which is implicated in the enhanced resistance to genotoxic stress, at least in human prostate and lung cancer cell lines [16]. The interplay between EGR-1 and p53 in gliomas may therefore be of high relevance to both tumor progression and drug resistance. Cisplatin is one of the most effective chemotherapeutic agents to date used for the treatment of many malignancies, including glioma [17]. Cisplatin causes tumor cell death by direct DNA damage and by generating reactive oxygen intermediates. Recent findings have suggested that these two factors may be responsible for activating the *EGR-1 *promoter. It was concluded that *EGR-1 *promoter can be induced by cisplatin [18]. In addition, pre-clinical studies have shown that cisplatin may be synergic with temozolomide, an oral alkylating agent, which is now widely used in the standard treatment of newly diagnosed and recurrent malignant gliomas [19,20]. In view of these results, we have asked the question whether EGR-1 has any role in chemoresistance to cisplatin or other drugs in glioma primary cells *in vitro*, and if this is related to the p53 status of the tumor or to other genes whose activity is required for the proper cytotoxic response. To this end, we have examined the response of freshly derived primary cell lines of malignant glioma, each established in our laboratory from a different donor, to cisplatin and five other cytotoxic drugs of relevant use. We concluded that the levels of EGR-1 protein in each cell line from *wild-type *cases of glioma strongly correlate with sensitivity to cisplatin.

## Results

### Primary cell lines differ in their response to anti-tumor drugs

**Figure 1:**
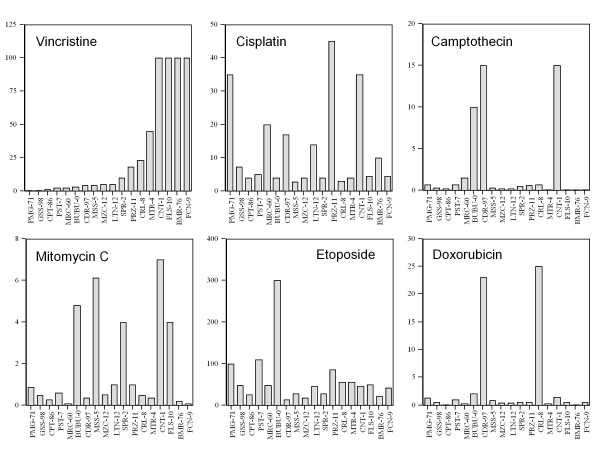
**Distribution of EC50 (μg/ml) index calculated for six anti-cancer therapeutic drugs in primary cell lines of human glioma**. Drug responses were evaluated with the acute cytotoxicity assay.

Eighteen glioma primary cell lines were each challenged with six cytotoxic drugs, namely vincristine, cisplatin, camptothecin, mitomycin C, etoposide (VP16), and doxorubicin, in the acute cytotoxicity assay (Figure 1). In the upper left quadrant, cell lines are ordered according to the concentration of vincristine needed to reduce by 50% (EC50) the number of living cells after three days of exposure, compared to the untreated control. Some of the cell lines (CNT-1, FLS-10, BMR-76, and FCN-9) were completely refractory. Therefore the EC50 could not be calculated. In such cases we provided the cell line with a EC50 value corresponding to the maximal administered dose which, in the case of vincristine, was 100 μg/ml. In the other quadrants cell lines are shown in the same order as for vincristine. Two cell lines (CDR-97, CNT-1) were resistant to camptothecin, one (BUBU-0) to etoposide, and one (CRL-8) to doxorubicin. As above, in these cases the assumed values of EC50 were 15, 300, and 25 μg/ml, respectively.

**Figure 2:**
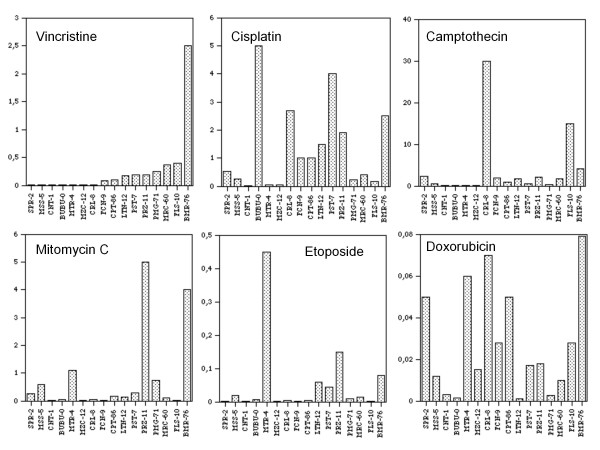
**Distribution of EC50 (μg/ml) index calculated for six anti-cancer therapeutic drugs in primary cell lines of human glioma**. Drug responses were evaluated with the clonogenic assay.

**Figure 3:**
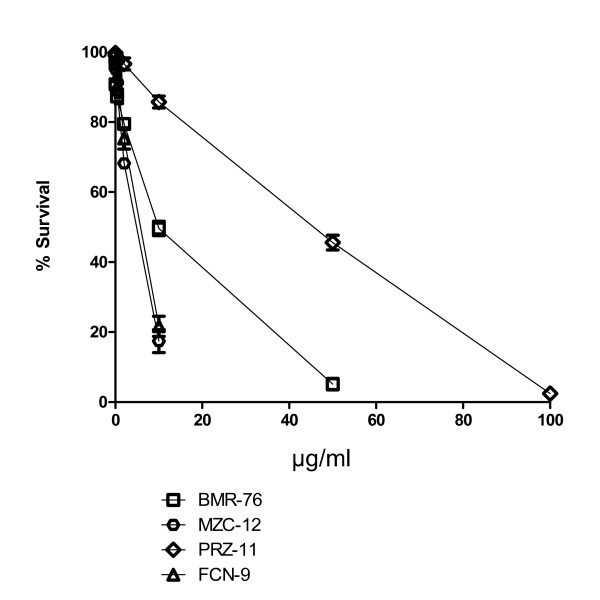
**Survival rates of four primary cell lines treated with different amounts of cisplatin**. Rates are referred to day 3 after treatment in the acute cytotoxicity assay.

The EC50 values corresponding to the drug concentration needed to lower by 50% the clonogenic activity of the cell lines are shown in Figure 2. Cell lines and EC50 values are presented with the same modality as in Figure 1. GSS-98 and CDR-97, since they are not clonogenic *in vitro*, could not be examined by the clonogenic assay and are not shown on the figure. According to the clonogenic assay, BMR-76 is resistant to vincristine, BUBU-0 to cisplatin, CRL-8 to camptothecin, and PRZ-11 to mitomycin C. In these cases we assumed as EC50 the values of 2.5, 5, 30, and 5 μg/ml, respectively. Most cell lines show wide ranges of response and different patterns of sensitivity. As an example, we show four representative cases of cell lines with different levels of sensitivity to cisplatin, translating into more (PRZ-11, MZC-12) or less (BMR-76, FCN-9) variable rates of survival when challenged with different drug amounts (Figure 3).

For each drug tested the mean, median, minimum, and maximum EC50 values are shown in Table 1 according to the acute cytotoxicity or the clonogenic assay. The ratio between the minimum and the maximum EC50 values for each drug is also reported. Except for doxorubicin, the EC50 values provided by the clonogenic assay range within a wider interval.

**Table 1:** Summary of cytotoxicity assay results

Clonogenic assay	Acute cytotoxic assay	Count	Mean	Median	Minimum	Maximum	EC50 ratio
Vincristine		16	0.264	0.082	0.0002	2.5	12,500
Cisplatin		16	1.330	0.760	0.020	5.0	250
Camptothecin		16	0.783	0.160	0.001	5.0	5,000
Mitomycin C		16	0.053	0.007	0.00004	0.450	11,250
Etoposide		16	3.843	1.333	0.016	30	1,875
Doxorubicin		16	0.028	0.018	0.001	0.079	79
							
	Vincristine	18	29.038	5.000	0.080	100	1,250
	Cisplatin	18	12.333	4.750	2.600	45	17.3
	Camptothecin	18	2.552	0.395	0.040	15	375
	Mitomicin C	18	1.782	0.550	0.050	7	140
	Etoposide	18	62.111	46.500	14.000	300	21.4
	Doxorubicin	18	3.253	0.535	0.150	25	166.7

### Primary cell lines are more sensitive to cisplatin when the frequency of EGR-1 positive cells in the tumor is lower

**Figure 4:**
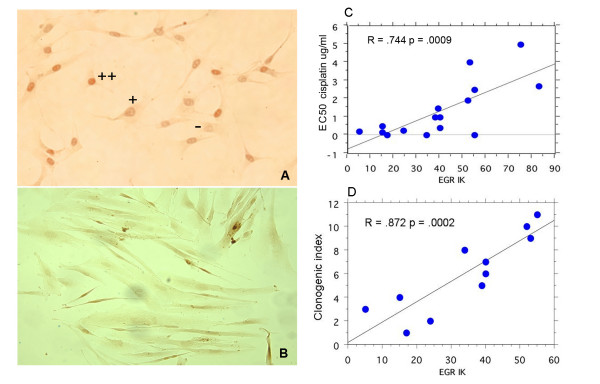
**EGR-1 expression by immunoistochemistry and response to anticancer drugs in primary cell lines of glioma**. A) BMR-76 primary cell line showing EGR-1 positive cells with different levels of positivity detected by an anti-EGR-1 polyclonal antibody. B) FLS-10, a mostly negative primary cell line. C) Relationship between the percentage of EGR-1 positive cells measured by immunoistochemistry and the EC50 response to cisplatin, measured with the clonogenic assay. D) Relationship between the clonogenic index and the percentage of EGR-1 positive cells measured by immunocytochemistry, in primary cell lines of human glioma carrying wild type copies of *p53*, *p16/INK4a *and *MDM2 *genes. This index is a global measure of the sensitivity to the six anticancer drugs measured with the clonogenic assay.

All eighteen primary cell cultures have been investigated in parallel for EGR-1 expression by immunofluorescence (IF), immunocytochemistry (IK) (Figure 4A,B) and western blot (WB). EGR-1 IF and IK positive cell lines are quantitated as percent positive cells, and are partially correlated to each other (R = .475, p = .0453). More significant is the correlation between EGR-1 IK (Figure 4A, B) and WB values (R = .703, p = .0007) [14]. For the further analysis, we have taken the values from immunocytochemistry as representative of EGR-1 expression. Percent IK positive cells range from 5% in the case of PMG-71 to 83% for CRL-8 (see below in Figure 4C). We then examined the correlation between the fraction of EGR-1 positive cells and the EC50 values seen for each drug. EGR-1 expression correlates significantly (R = .744, p = .0009) with sensitivity to cisplatin measured by the clonogenic assay (Figure 4C).

### EGR-1 expression, mutations in oncosoppressive genes and drug cytotoxicity

Six out of 18 cell lines have mutations in the *p53 *gene or in the *p16/INK4a/ARF *gene complex. None has amplified copies of *mdm2 *gene. Thus, CRL-8 has a homozygous mutation at codon 216 (Val→Met), FCN-9 at codon 224 (ag→aa), MZC-12 and BUBU-0 at codon 248 (Arg→Glu) of p53 gene. FLS-10 and GSS-98 present a homozygous deletion at the *p16/INK4a/ARF *locus [14]. Thus, concerning the relationship between drug sensitivity and EGR-1 expression, we asked the question whether the presence of the above mutations in these cell lines may affect their response. After dividing the cell lines into two groups, mutated and not-mutated, we found that sensitivity to cisplatin correlates significantly with the percentage of EGR-1 positive cells only in cell lines without mutations (R = .696, p = .0173). In addition to cisplatin we found that the levels of EGR-1 expression in these cell lines also correlated significantly with sensitivity to Mitomycin C, as seen by the acute cytotoxicity assay (R = .588, p = .0444). The mean value of EC50 to each drug was then calculated for the groups of mutated and not-mutated cell lines, and compared with each other (Table 2). Differences between mutated (column D) and not-mutated (column C) cell lines were found for the response to camptothecin (1.127 vs. 0.025, p = 0,007), mitomycin C (0.075 vs. 0.002, p = 0.026) and etoposide (1.312 vs. 9.410, p = 0.052), all cases measured by the clonogenic assay. Mutated cell lines have higher values of EGR-1 (54.66% vs. 35.33%). In addition, we divided the group of not-mutated cell lines into two further subgroups of six cell lines each, with low and high EGR-1 values, respectively, and compared their mean EC50 (Table 2). The two not-mutated groups with lower and higher EGR-1 differed significantly in their mean EC50 for vincristine (0.069 vs. 0.664; p = 0.044) and cisplatin (0.427 vs. 1.960; p = 0.034), measured by the clonogenic assay, and for mitomycin C (3.218 vs. 0.413; p = 0,040) by the acute citotoxicity assay.

**Table 2:** Comparison of EC50 mean values between mutated and not-mutated cell lines.

		Cell lines without mutations			Cell lines with mutations
		**A**	**B**	**C**	**D**

		**Lower** **IK EGR-1** **n = 6**	**Higher** **IK EGR-1** **n = 6**	**Total** **n = 12**	**Total** **n = 6**

		X^a ^= 22.33	X^a ^= 48.33	X^a ^= 35.33	X^a ^= 54.66

Vincristine	C^b^	**0.069**	**0.664**	0.339	0.098
	
	A^c^	27.31	21.21	24.27	38.58

Cisplatin	C	**0.427**	**1.960**	1.124	1.784
	
	A	15.73	16.83	16.3	4.433

Camptothecin	C	0.472	1.914	**1.127**	**0.025**
	
	A	2.783	2.992	2.887	1.881

Mitomycin C	C	0.089	0.09	**0.075**	**0.002**
	
	A	**3.218**	**0.413**	1.82	1.715

Etoposide	C	0.819	1.904	**1.312**	**9.410**
	
	A	50.16	50.66	50.41	85.50

Doxorubicin	C	0.021	0.035	0.027	0.028
	
	A	0.758	4.180	2.47	4.820

EC50 values were compared between column A and B, and between column C and D.

Statistically different EC50 values are in **bold **(see inside text).

X^a ^= IK EGR-1 mean value

^b ^= clonogenic assay, ^c ^= acute cytotoxicity assay

Finally, we elaborated an index for ranking the cell lines according to their sensitivity by integrating their responses into an overall cumulative score based on the results obtained with the acute cytotoxic assay and the clonogenic assay, respectively. The clonogenic ranking index (Figure 4D) correlates significantly with the values of EGR-1 expressed in the not-mutated cell lines (R = 0.872, p = 0.0002).

### Tumorigenic expression and drug sensitivity in glioma primary cell lines

**Figure 5:**
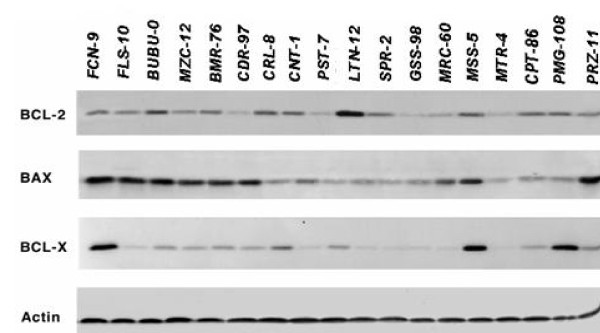
**Western blot of BCL-2, Bax and BCL-X in glioma derived primary cell cultures**. β-Actin was used as loading control.

We investigated the expression of other tumor-related products, in addition to EGR-1, which might be influencing the response of the tumor cells to the anti-neoplastic treatment. We calculated by immunofluorescence the percentage of cells carrying the expression of MDM2, GFAP, and p21, and by western blot the production of fibronectin, Bcl-2, Bcl-xL, Bax (Figure 5) and TGF-β. We then assessed whether there was an association between drug sensitivity and these other parameters. We found that the percentage of MDM2 positive cells correlates significantly with the response to doxorubicin in the acute assay (R = .619, p = .0062) and to vincristine in the clonogenic assay (R = .504, p = .0466), and that the production of TGF-β correlates significantly with the response to etoposide in the acute assay (R = .517, p = .0336). These correlations refer to the whole group of cell lines. As to the subgroup of cell lines not carrying mutations in the *p53 *gene or the *p16/INK4a/ARF *gene complex, we again found statistically significant correlations between the percentage of MDM2 positive cells and the response to doxorubicin (R = .79 with p = .0022, and R = .765 with p = .0061) and to vincristine (R = .569 with p = .0535, and R = .644 with p = .0325) in the acute cytotoxicity and the clonogenic assay, respectively, between the response to VP16 and the production of both TGF-β (R = .609, p = .0357) and Bax (R = .764, p = .0457), the production of fibronectin and the response to cisplatin (R = .826, p = .0116) and to camptothecin (R = .732, p = .0389) in the clonogenic assay and, finally, between the percentage of GFAP positive cells and the response to Mitomycin C in the clonogenic assay (R = .896, p = .0002). This last association has clearly been the most significant.

## Discussion

Resistance to anti-neoplastic drugs is certainly one of the most important factors that limits the progress of current therapy for cancer. Chemoresistance in glioma is based on a complex network of multiple pathophysiological mechanisms such as altered functioning of membrane pumps, poor tumor perfusion or insufficient blood vessel supply, to cite only a few [21,22]. At molecular level, one of the most investigated factors is represented by p53. Although p53 is a crucial apoptotic cell death mediator in cancer cells following genotoxic stress, its direct role in chemosensitivity of gliomas is still controversial. In fact, studies *in vitro *of the p53 genetic or functional status of glioma cell lines failed to predict in either acute cytotoxicity or clonogenic cell death assays the response to anticancer drugs [23]. It was concluded by many that the role of p53 in tumor therapy is complex, and its status cannot be taken as a predictive tool. Most of the well characterized established cell lines from glioma have run through countless passages *in vitro*. As a result, they must have accumulated both chromosomal and gene mutations, aside from the genetic alterations present in the tumor from which they were derived. Secondly, they all have mutated copies of *p53*, *p16*, or *mdm2 *genes and thus have an altered p53 pathway. At variance with these reports, in our study we worked with freshly derived primary cell lines, which replicated for a very limited number of passages. Only 6 cell lines out of 18 have mutations occurring at the *p53 *or *p16 *genes, and none had mutations at the *mdm2 *gene locus. We could therefore compare our assay results between p53 mutated and not-mutated cell lines. We focused on the question whether EGR-1 expression might be related to chemosensitivity of the tumor cells *in vitro*. EGR-1 has multiple roles that might affect cell sensitivity to antiblastic drugs. In fact, EGR-1 is responsible for regulating cell proliferation and the response to several types of stress stimuli, including the apoptotic response. For these properties and for being deregulated in human gliomas, as in other tumors, we sought to look for correlations between the levels of EGR-1 expression in our cell lines and their sensitivity to genotoxic anticancer drugs. We found that EGR-1 expression levels did in fact correlate with response to cisplatin: the lower is EGR-1 expressed, the higher is the cytotoxic response. In conclusion, EGR-1 emerged as a predictor of chemoresistance for cisplatin. We found that EGR-1 levels correlate significantly with sensitivity to cisplatin both in cell lines without mutations in the p53 pathway and in the whole group, and that this applies also to several other drugs, though to a lesser extent. The same is not true for the six cell lines with mutated p53. These results are in agreement with the general view that mutations affecting the p53 response pathway act unfavorably by accelerating the progression of the disease and possibly by affecting the cytotoxic response to drugs. That tumors are more sensitive to the anti-neoplastic action of drugs when EGR-1 is less expressed is compatible with the protective properties of EGR-1 as anti-stress agent, and with the fact that EGR-1 down-regulation is likely to be a prerequisite for the growth of tumors harbouring intact copies of p53 gene. We also found a correlation between MDM2 expression and drug sensitivity. In fact, both the sensitivity to doxorubicin and to vincristine increase their correlation with the percent of MDM2 positive cells when they are assessed in cell lines which carry only wild-type copies of the *p53 *gene, from .62 to .79 in the case of doxorubicin assessed with the acute cytotoxicity assay, and from .50 to .64 for vincristine assessed with the clonogenic assay. We interpreted these results according to the view that when p53 is functional, an increase in the fraction of MDM2 positive cells should parallel an increase in the fraction of cells where p53 becomes inactive. This is in contrast with past evidence that there is no role for the levels of p21 or MDM2 proteins as a predictor of response to chemotherapeutics in glioma cell lines [24,25].

## Conclusion

The results of chemotherapy in gliomas are largely disappointing. In fact, even temozolomide which is the gold standard today for first-line therapy of glioblastomas, is ineffective in resistant primary gliomas and in most of the recurrent malignant cases, for which at present there are no chemotherapeutic regimens. A better knowledge of the molecular markers for chemosensitivity could help us to identify the cases which are sensitive to the most used chemotherapeutics. Our results provide new preliminary evidence for the role of EGR-1 as a molecular factor involved in the chemosensitivity of glioma. Several authors have already indicated that resistant and recurrent gliomas can be alternatively treated with cisplatinum in combination with other drugs [19,26]. Validation of these observations on a larger scale, together with functional experiments, could pave the way toward the elucidation of new molecular aspects of pathophysiology of drug resistance and the definition of a sensitive tool for the prediction of a successful therapy.

## Materials and methods

### Glioma primary cell cultures

The research protocol was drafted in accordance with the Declaration of Helsinki, reviewed and approved by an institutional board. Written informed consent for research use of tumor tissue was obtained from each patient prior to surgery. Primary cell lines were established from anaplastic astrocytoma or glioblastoma multiforme tissue obtained at surgery and diagnosed according to the W.H.O. classification. Cells were cultured in Dulbecco's modified Eagle medium containing 10% fetal calf serum and 2% glutamine. We used fresh cells collected from passage 3 to 8, or cells preserved in liquid nitrogen from earlier passages. Several cell lines have been reported in a previous paper [14].

### Anti-tumor drugs

Six cancer therapeutic drugs have been tested in our assays: a mitotic inhibitor, vincristine; two cross-linkers of DNA, cisplatin and mitomycin C; an anthracyclin antibiotic which intercalates DNA, doxorubicin; an inhibitor of the DNA enzyme topoisomerase I, camptothecin; and an inhibitor of the enzyme topoisomerase II, etoposide. They were all purchased from Sigma Inc. (St. Louis, MO, USA). Drugs were dissolved at 100 times the final concentration tested, and sterilized by filtration before use. Stock drugs solutions were made in absolute ethyl alcohol (vincristine), in 10 mM dimethylsulfoxide (mitomycin C, cisplatin, camptothecin, etoposide), or in water (doxorubicin). Vincristine and doxorubicin were each time freshly prepared, cisplatin was stored at room temperature, all the others at -20 C.

### Drug sensitivity

For the acute cytotoxicity assay, the glioma cells were seeded at 5 × 10^3 ^cells per well in 96-well plates, allowed to attach for 24 h and subsequently exposed to drugs for 72 h in triplicate wells. Cell survival was determined using a colorimetric MTT (3-(4,5-dimethylthiazol-2-yl)-2,5-diphenyl tetrazolium bromide) assay [27]. Fifty μl of 5 mg/μl MTT was added to each well and incubated for 4 h at 37°C. The absorption was read at 540 nm using an automated microplate reader. For the clonogenic assay, cells were seeded at 0.5 × 10^3 ^cells per well in 96-well plates, allowed to attach for 48 h, exposed to the drugs for 24 h, washed, and allowed to grow for 2 to 3 weeks in drug-free medium. The control wells were carefully monitored not to reach confluence during these assays. Growth was measured by crystal violet assay [27]. The colonies were stained, and the dye was subsequently released by citrate buffer for quantification in an ELISA reader. Results were given as the drug concentration dose which affected cell growth by 50% compared to untreated cultures (EC50, μg/ml). The EC50 value is obtained by linear interpolation of growth values of cells following treatment with a minimum of four different doses.

### Western Blot Analysis

Bcl-2, Bcl-xl and Bax, were all detected in 100 μg of protein from whole cell extracts, according to the procedure described in [9,10]and actin was used as loading control. Protein concentration was estimated using BioRad assay kit. Primary and secondary antibodies were purchased from Santa Cruz Biotechnology (Santa Cruz, Ca USA). Relative measures of protein expression were obtained through densitometric analysis of Western blot chemiluminescent bands using the public domain NIH Image software (developed at the U.S. National Institutes of Health) and normalizing with respect to the actin content of each sample.

### Immunofluorescence and immunocytochemistry

For EGR-1, we stained the cells with the same primary antibody used in western blot experiments. Immunofluorescence and immunocytochemistry were performed on cells fixed in 4% fresh paraformaldehyde. Cells were also stained with a mouse monoclonal antibody against GFAP (Sigma), and mouse monoclonal antibodies against p21 and MDM2 (Pharmingen Corporation, San Diego CA, USA). Immunocytochemistry was performed using avidin-biotin-peroxidase (ABC Universal kit, Vector Laboratories, Burlingame CA, USA) following the manufacturer's protocol. Values are expressed as percentage of positive cells stained.

### Analysis of p53 mutations

p53 mutations in exons 5 to 9 were sequenced as described in [9,25]. Sequencing was performed using a Big Dye terminator DNA sequencing kit with the ABI PRISM 377 DNA Sequencer (PE Applied Biosystems Inc., Foster City CA, USA), according to the manufacturer's instructions.

### Assessment of p16 and MDM2 status

Analysis of allele dosage for MDM2 and loss of heterozygosity at the *p16/INK4a/ARF *locus were performed using procedures and probes as previously reported [9].

### Statistical analysis

For comparisons of two groups, a *t*-test for independent samples or non parametric Mann-Whitney U test were used. Correlations were computed by Pearson correlational analysis. Statistical analysis and calculation of the regression coefficients were performed using StatView software (SAS Institute Inc., Cary NC, USA).

For calculating the acute cytotoxicity and the clonogenic index, we first assigned for every cell line a ranking value for each of the administered drugs. One was the value assigned to the most sensitive and 18 to the most resilient, with 2 to 17 being all the other intermediate values. The six values obtained from each cell line in either assay were added together, and the new values thus obtained were used as basis for the final ranking of the cell lines. For ease of evaluation of the results, these values (acute cytotoxicity index and clonogenic index) were changed into the values from 1 to 18.

## Abbreviations

EGR-1: early growth response gene 1; ARF: ADP-ribosylation factor; TGF-β: transforming growth factor beta; PTEN: phosphatase and tensine homologue gene;

## Competing interests

The authors declare that they have no competing interests.

## Authors' contributions

AC and GR have made substantial contributions to the conception and design of experiments and the drafting of the manuscript. AP carried out the molecular studies. CF performed the statistical analysis. VL carried out immunofluorescence and immunocytochemistry. AA and MM carried out western-blot analysis. DP participated in interpretation of data and she was involved in the drafting of the manuscript. All authors read and approved the final manuscript.
